# Risk factors of impaired glucose tolerance in obese children aged 12-15 years old

**DOI:** 10.1186/1687-9856-2013-S1-P98

**Published:** 2013-10-03

**Authors:** Francisca Yohanna Maria Kristiani

**Affiliations:** 1 Division Of Pediatric, Cipto Mangunkusumo Hospital, Jakarta Pusat, Indonesia

## Background

Report of obesity in children is increasing up to the alarm rate. Impaired glucose tolerance (IGT) is a risk factor for type 2 diabetes mellitus (T2DM) in obese children. The last researches described different prevalence of IGT in many region. However, study regarding the risk factors of IGT in Indonesian child is still limited.

## Objectives

To identify risk factors of IGT in children 12-15 years old.

## Methods

A cross sectional study was performed in 12-15 years old obese children from 10 Junior High Schools in Jakarta. History of T2DM in the family was determined by anamnesis with the parent. Clinical sign of acanthosis nigricans (AN) was fixed by physical finding. The intensity of physical activity was measured using the activity criteria according to World Health Organization*.* The diagnosis of IGT was determined using an oral glucose tolerance test (OGTT) according to American Diabetes Association (ADA) criteria in 2006. Proportion of AN, history of T2DM in the family, physical activity, and IGT were measured. Fisher test was used to analyse the risk of IGT based on BMI; Mann-Whitney test was based on AN, physical activity, and history of T2DM. Logistic regresion analysis was done based on the clinical judgement.

## Results

There was 182 patients with an equal distribution among sex. Proportion of IGT and AN respectively was 3.8% and 93.9%. A total of 56.7% patients with AN was in the range of 30-39.9 for BMI, and all patients in the BMI range of 40-49.9 had severe AN (grade 3-4). All subjects who had IGT had severe degree of AN (grade 4) and none was active physically. Family history of T2DM was found in 57.1% subjects with IGT, and 46.9% without IGT. Active physical activity was found in 25.1% subjects without IGT. There were no correlation between AN severity, history of T2DM in the family, intensity of physical activity, and BMI with the incidence of IGT (p =1; 0.7; 0.2; and 0.9, respectively). Logistic regression analysis found that all independent variables were not a risk factor for the incidence of IGT (p> 0.05).

## Conclusion

There was no relationship between BMI, AN severity, history of T2DM in the family, and intensity of physical activity with the incidence of IGT. All subjects with IGT have fourth degree AN.

